# Development of an intramolecular charge transfer-type colorimetric and fluorescence sensor for water by fusion with a juloidine structure and complexation with boron trifluoride†

**DOI:** 10.1039/c9ra07136a

**Published:** 2019-10-03

**Authors:** Keiichi Imato, Toshiaki Enoki, Yousuke Ooyama

**Affiliations:** Department of Applied Chemistry, Graduate School of Engineering, Hiroshima University 1-4-1 Kagamiyama Higashi-Hiroshima 739-8527 Japan yooyama@hiroshima-u.ac.jp

## Abstract

An optical sensor with the ability to detect and determine water over a wide concentration range is highly desirable in the laboratory and industry. Here the sensitivity and spectral responses of an intramolecular charge transfer-type colorimetric and fluorescence sensor with β-carboline structure are tuned and improved significantly over various water contents in the organic solvent by fusion with an electron-donating juloidine structure and complexation with boron trifluoride (BF_3_). The sensors, ET-1 and ET-1-BF_3_, developed in this study can respond differently depending on water content. ET-1-BF_3_ releases BF_3_ to generate ET-1 by addition of a trace amount of water, and ET-1 forms hydrogen bonds with one water molecule in low water contents and a hydrogen-bonded proton transfer complex with several water molecules in high water contents, accompanying gradual color and fluorescence changes. This work shows a promising approach to the sensitive detection and precise determination of water over the whole concentration range using a simple and practical method with optical sensors.

## Abstract 2

An intramolecular charge transfer-type optical sensor fused with a juloidine structure and complexed with boron trifluoride can detect and determine water over a wide concentration range.

## Graphical abstract

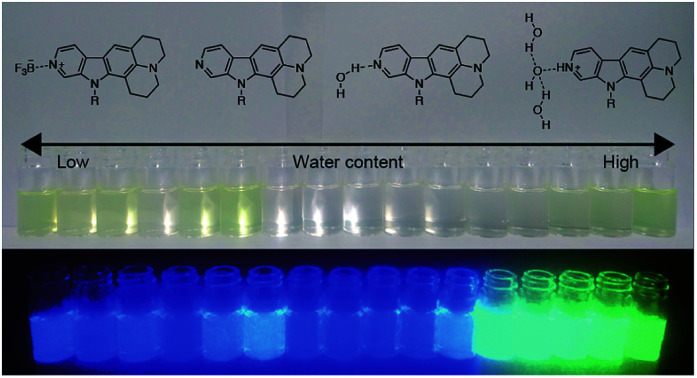

## Introduction

The detection and determination of water are of great importance in industrial applications, including food inspection, biomedical and environmental monitoring, and manufacturing of pharmaceutical, electronic, and petroleum products.^1^ In synthetic chemistry, the presence of water in organic solvents causes serious problems, such as generation of by-products, quenching of reactions, lowering of the product yields, and furthermore, catastrophic dangers of fire and explosion. Particularly, in a large-scale industrial process, careful attention is paid to this impurity to avoid worst-case scenarios. Therefore, various analytical approaches and techniques have been developed to detect and determine water content in organic solvents. The standard Karl Fischer titration is the most popular method and allows for quantitative measurements of water over a wide range of the concentrations (0.001–100% water) with high sensitivity and wide applicability for a large variety of samples;^2,3^ however, it has several limitations, *e.g.*, instability, time-consuming procedure, and use of toxic chemical reagents and costly specialized instrumentation. Therefore, in recent years, the method using optical water sensors has attracted much attention because of its considerable advantages of simple operation, low-cost fabrication, fast response, high sensitivity, non-destructive nature, and remote and *in situ* monitoring even by the naked eye.^4^ Although most research studies on the optical sensors have been focused on the detection and determination of a trace amount of water,^5–28^ sensors that can work over a wide range of water concentrations (similar to the range the Karl Fischer method can cover) are also required.

In the optical sensor method, water content can be determined by measurement and ratiometric analysis of the optical properties of small-quantity sensor molecules doped in sample solutions, *i.e.*, wavelength, intensity, lifetime, and quantum yield of photoabsorption and photoluminescence. Therefore, the optical properties should be sensitive to water molecules and variable depending on the water content. Several strategies have been demonstrated to meet the requirement, such as intramolecular charge transfer (ICT),^5–9^ excited state intramolecular proton transfer (ESIPT),^9–11^ photo-induced electron transfer (PET),^12–18^ aggregation-induced emission (AIE),^7,29,30^ decoloration of hydrogen-bonding photochromic dyes,^31^ and other water-triggered mechanisms.^19–28^ Generally, in each system, there is a specific range of water concentration available for detection; AIE is observed in high water concentrations,^7,29,30^ whereas other systems exhibit optical changes in a relatively low concentration range.^5–28^ In this context, a reasonable approach to incorporating two mechanisms into one sensor molecule has been developed to detect and determine water in organic solvent sensitively in a wide concentration range, including the combinations of ICT or PET and AIE,^32–39^ two water-induced reactions,^40^ and two ICT-related mechanisms,^41,42^ with rare exceptions based on one mechanism.^23,43–49^ However, the pioneering systems still have several problems to be solved, such as the appearance of non-response ranges to variations in water content and the indiscernible small changes in the photoabsorption and fluorescence spectra as well as by the naked eye.

**Fig. 1:**
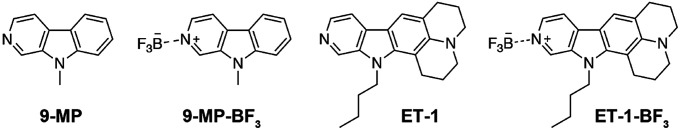
Chemical structures of 9-MP, 9-MP-BF_3_, ET-1, and ET-1-BF_3_.

Herein, we report the tuning of sensitivity of optical sensors to water molecules and improvement in the spectral variations over a wide range of water content in an organic solvent. We focused on the β-carboline-based water sensor, 9-MP,^50^ with a small electron donor–acceptor (D–A) structure and its complex with boron trifluoride (BF_3_), 9-MP-BF_3_,^42^ as shown in Fig. 1, because they exhibit a unique multi-step response to increasing water content in organic solvent, *i.e.*, release of BF_3_ (only in 9-MP-BF_3_), hydrogen bonding with one water molecule, and formation of hydrogen-bonded proton transfer complex (PTC)^50–58^ with several water molecules, accompanying gradual spectral changes in photoabsorption and fluorescence based on the D–A structure and ICT. However, these systems also lack the capability to detect and determine water in moderate concentrations (*ca.* 10–40 wt%) and show the indiscernible optical changes particularly in photoabsorption spectra also in the other ranges.^42,50^ Since the unique response is owing to the moderate basicity of the pyridinic nitrogen atom in the β-carboline skeleton, in this study, we modulated the basicity and enhanced the D–A and ICT characteristics by fusion of the β-carboline skeleton with a strong electron donor, juloidine,^59^ developing new water sensors, ET-1 and ET-1-BF_3_. Their water-sensing ability and sensing mechanisms were investigated in acetonitrile with various water contents, and the strategy for tuning of the sensitivity to water and improvement in spectral response over a wide range of water content were demonstrated.

## Experimental

### General methods

Melting points were measured using a Yanaco micro melting point apparatus MP model. IR spectra were recorded on a Shimadzu IRAffinity-1 spectrometer using the ATR method. High-resolution mass spectral data were acquired using a Thermo Fisher Scientific LTQ Orbitrap XL. ^1^H NMR, ^13^C NMR, and ^11^B NMR spectra were recorded using Varian-400 (400 MHz) and Varian-500 (500 MHz) FT NMR spectrometers. Photoabsorption spectra were recorded using a Shimadzu UV-3150 spectrophotometer and fluorescence spectra were recorded using a HITACHI f-4500 fluorescence spectrometer. The determination of water in acetonitrile solution was done with MKC-610 and MKA-610 Karl Fischer moisture titrators (Kyoto Electronics manufacturing Co., Ltd.) based on Karl Fischer coulometric titration (relative standard deviation is below 0.3% in a measurement of methanol containing 1 mg water) for below 1.0 wt% and volumetric titration for above 1.0 wt%, respectively.

### Synthesis of 13-butyl-2,3,5,6,7,13-hexahydro-1*H*-pyrido[3,2,1*ij*]pyrido[4′,3′:4,5]pyrrolo[2,3-*f*]quinoline-boron trifluoride complex (ET-1-BF_3_).

To a solution of ET-1 (ref. 59) (0.03 g, 0.09 mmol) in 6 mL of Et_2_O was added dropwise 47% BF_3_–OEt_2_ (0.09 mmol) diluted with ether (2 mL) for 30 min, and then, the solution was stirred for 4 h at room temperature. The resulting precipitate was filtered and was washed by Et_2_O to give ET-1-BF_3_ (0.03 g, 43% yield) as a yellow solid; mp: 157–158 °C; IR (ATR): *ν̃* = 1611, 1437, 1030 cm^−1^; ^1^H NMR (400 MHz, CD_3_CN): *δ* = 0.98 (t, *J* = 7.4 Hz, 3H), 1.38–1.42 (m, 2H), 1.72–1.82 (m, 2H), 2.00–2.04 (m, 4H, overlapping peak of residual proton in CD_3_CN), 2.88 (t, *J* = 6.2 Hz, 2H), 3.20 (t, *J* = 6.4 Hz, 2H), 3.31–3.38 (m, 4H), 4.48 (t, *J* = 7.8 Hz), 7.66 (s, 1H), 7.95 (d, *J* = 6.2 Hz, 1H), 8.08 (d, *J* = 6.2 Hz, 1H), 8.51 (s, 1H) ppm (one aliphatic proton signal was not observed owing to overlapping resonances); ^13^C NMR (125 MHz, CD_3_CN): *δ* = 13.96, 20.57, 21.68, 22.22, 23.99, 46.85, 50.36, 51.77, 102.23, 110.82, 113.29, 121.40, 121.41, 121.83, 129.62, 135.69, 137.28, 145.37, 148.99 ppm; ^11^B MNR (160 MHz, CD_3_CN): *δ* = −1.13 ppm; HRMS (ESI): *m*/*z* (%): [M + H^+^] calcd for C_22_H_25_N_3_, 320.21212, found 320.21240.

## Results and discussion

The juloidine-conjugated β-carboline complexed with BF_3_, ET-1-BF_3_, was effectively prepared by treating the corresponding β-carboline derivative, ET-1, with BF_3_–OEt_2_ and characterized by ^1^H NMR, ^13^C NMR, ^11^B NMR, and FT-IR measurements and high-resolution mass analysis.

**Fig. 2:**
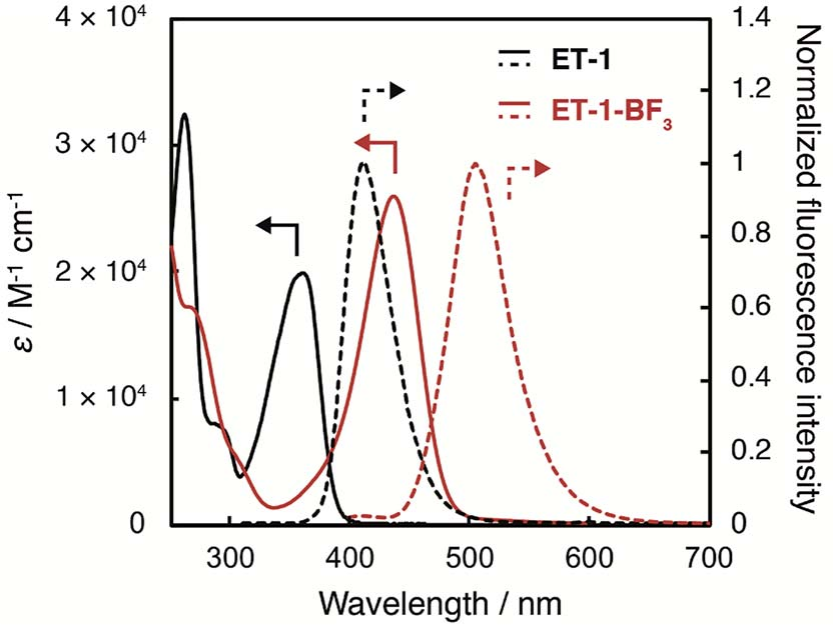
Photoabsorption and fluorescence spectra of ET-1 and ET-1-BF_3_ in acetonitrile (*λ*^ex^ = 310 and 382 nm for ET-1 and ET-1-BF_3_, respectively).

The photoabsorption and fluorescence spectra of ET-1 and ET-1-BF_3_ in acetonitrile are shown in Fig. 2. The photoabsorption maxima (*λ*^abs^_max_) of ET-1 and ET-1-BF_3_ were observed at 365 and 436 nm, respectively, which originate from the ICT excitation from the electron-donating julolidine moiety to the electron-withdrawing pyridine moiety in ET-1 or to its complex with BF_3_ in ET-1-BF_3_. Because the strong electron-withdrawing ability of the pyridine complex with BF_3_ enhances the ICT characteristic, the photoabsorption maximum of ET-1-BF_3_ was observed at longer wavelength region by 71 nm than that of ET-1. Additionally, the molar excitation coefficient value (*ε*) of ET-1-BF_3_ (25 900 M^−1^ cm^−1^) was higher than that of ET-1 (19 900 M^−1^ cm^−1^). Similarly, the corresponding fluorescence bands of ET-1 and ET-1-BF_3_ appeared at 411 and 507 nm (*λ*^fl^_max_), respectively, and the fluorescence quantum yields (*Φ*_f_) were 0.38 (ET-1) and 0.65 (ET-1-BF_3_). These results indicate that the complexation of ET-1 with BF_3_ caused not only the bathochromic shifts of the photoabsorption and fluorescence bands but also the increases in *ε* and *Φ*_f_ values. Furthermore, compared with 9-MP and 9-MP-BF_3_ in acetonitrile,^42,59^ the fusion with juloidine could improve their photophysical properties (*λ*^abs^_max_ = 358 and 388 nm, *ε* = 5800 and 3300 M^−1^ cm^−1^, *λ*^fl^_max_ = 368 and 458 nm, and *Φ*_f_ = 0.04 and 0.52 for 9-MP and 9-MP-BF_3_, respectively).

**Fig. 3:**
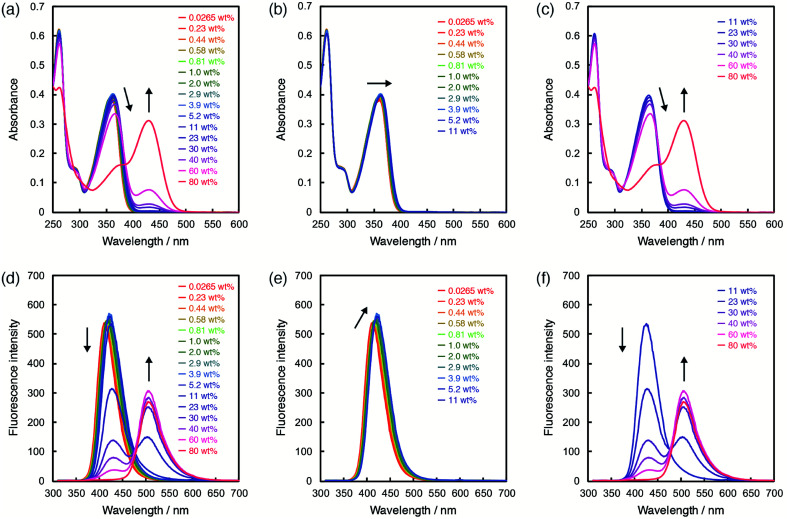
Photoabsorption spectra of ET-1 (*c* = 2.0 × 10^−5^ M) in acetonitrile containing (a) 0.0265–80 wt%, (b) 0.0265–11 wt%, and (c) 11–80 wt% of water. Fluorescence spectra of ET-1 (*c* = 2.0 × 10^−5^ M, *λ*^ex^ = 302 nm) in acetonitrile containing (d) 0.0265–80 wt%, (e) 0.0265–11 wt%, and (f) 11–80 wt% of water.

**Fig. 4:**
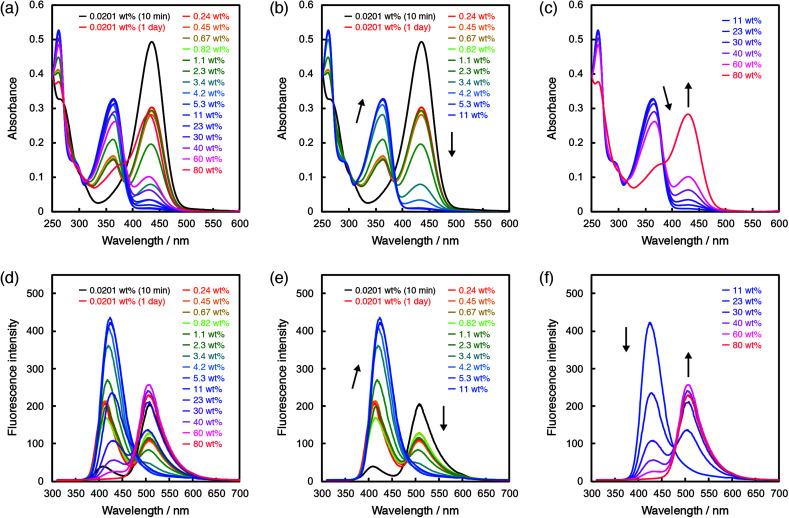
Photoabsorption spectra of ET-1-BF_3_ (*c* = 2.0 × 10^−5^ M) in acetonitrile containing (a) 0.0201–80 wt%, (b) 0.0201–11 wt%, and (c) 11–80 wt% of water. Fluorescence spectra of ET-1-BF_3_ (*c* = 2.0 × 10^−5^ M, *λ*^ex^ = 302 nm) in acetonitrile containing (d) 0.0201–80 wt%, (e) 0.0201–11 wt%, and (f) 11–80 wt% of water.

In order to investigate the ability of ET-1 and ET-1-BF_3_ as optical sensors for water in organic solvent, the photoabsorption and fluorescence measurements were performed in acetonitrile with various water contents (Fig. 3 and 4). In the case of ET-1, the photoabsorption band at around 360 nm slightly increased in intensity and bathochromically shifted with increase in water content up to 11 wt%, and two isosbestic points were observed at 298 and 354 nm (Fig. 3b). These small spectral changes can be ascribed to the formation of the hydrogen-bonded complex (ET-1-H_2_O) between the pyridinic nitrogen atom and one water molecule.^42,50–58^ Above 11 wt%, the photoabsorption band decreased in intensity and red-shifted distinctly, while a new photoabsorption band appeared simultaneously at around 430 nm (Fig. 3c). The new band is assignable to the ICT band of the hydrogen-bonded PTC (ET-1-H^+^) formed by the proton transfer from water molecule to the pyridinic nitrogen atom. Since the photoabsorption spectra of 9-MP changed negligibly over the whole range of water content,^42,50^ these results indicate that the fusion of juloidine and the enhanced ICT characteristic improved its spectral responses to water. Similar behavior was also observed in the fluorescence spectra of ET-1. The solution showed a small red-shift and increase in intensity of the fluorescence band at around 420 nm by addition of water up to 11 wt% (Fig. 3e), indicating the formation of the hydrogen-bonded complex (ET-1-H_2_O). From 11 wt%, the intensity of the fluorescence band started decreasing significantly, while a new band originating from the hydrogen-bonded PTC (ET-1-H^+^) simultaneously appeared at around 510 nm and increased in intensity with an isoemissive point at 475 nm (Fig. 3f). In the previous study,^59^ these spectral changes were observed by not increasing the static dielectric constant of solvent but changing the solvent to hydrogen-bonding protic one. From these results, we concluded that the hydrogen-bonded complex (ET-1-H_2_O) started forming from a trace content of water, followed by the simultaneous generation of the hydrogen-bonded PTC (ET-1-H^+^) above 11 wt%. Additionally, it is noteworthy that ET-1 started forming the hydrogen-bonded PTC from the lower water content (11 wt%) compared to 9-MP (from 40 wt%)^42,50^ due to the enhanced basicity of the pyridinic nitrogen atom in ET-1 and resulting promotion of the PTC formation, indicating a successful tuning of the sensitivity over a wide range of water content.

In the case of ET-1-BF_3_, the photoabsorption and fluorescence spectral responses to water were complicated. First, we examined the time dependence of the spectra after addition of a tiny amount of water. In the photoabsorption and fluorescence spectra, large differences were observed between the ET-1-BF_3_ acetonitrile solutions that were stored in the dark for 10 min or 1 day after addition of 0.0201 wt% of water (Fig. 4a and d). The longer storage time caused significant decreases in intensity of the photoabsorption band at around 430 nm and fluorescence band at around 510 nm and also increases in intensity of the photoabsorption band at around 360 nm and fluorescence band at around 420 nm. Because the time-dependent spectral changes are attributed to the water-induced release of BF_3_ and generation of ET-1,^41,42^ the results indicate that the reaction occurs with a trace amount of water but takes a relatively long time. Therefore, we employed the solutions stored in the dark for 1 day after adding various amounts of water for the following photoabsorption and fluorescence measurements. By the addition of water up to 11 wt%, the photoabsorption band at around 430 nm drastically decreased in intensity and almost disappeared with simultaneous increase of that at around 360 nm, which originates from ET-1 generated as a result of the BF_3_ release (Fig. 4b). From 11 wt%, the photoabsorption band at around 360 nm decreased in intensity, while that at around 430 nm reappeared and increased, indicating the formation of the hydrogen-bonded PTC (ET-1-H^+^) (Fig. 4c). The formation of the hydrogen-bonded complex (ET-1-H_2_O) is seen from the bathochromic shift of the photoabsorption band at around 360 nm in a wide range above 2.3 wt% (below 40 wt%) (Fig. 4b and c). In the spectra, isosbestic points were observed at 272 and 293 nm below 2.3 wt% and 272, 305, and 386 nm above 40 wt% of water content. In the corresponding fluorescence spectra (Fig. 4d–f), the intensity of the band at around 510 nm originating from ET-1-BF_3_ decreased with increase of that at around 420 nm originating from ET-1 below 11 wt% of water content (Fig. 4e). In the range, the band at around 420 nm red-shifted with increase in water content, indicating the simultaneous formation of ET-1-H_2_O.^42,50–58^ Above 11 wt%, the fluorescence band at around 420 nm decreased in intensity, while that at around 510 nm significantly increased with an isoemissive point at 475 nm, due to the generation of ET-1-H^+^ (Fig. 4f). From the comparison between Fig. 3 and 4, it can be concluded that the sensitivity and spectral response to water could be further improved in the low water contents by the complexation of the juloidine-conjugated β-carboline sensor (ET-1) with BF_3_.

**Fig. 5:**
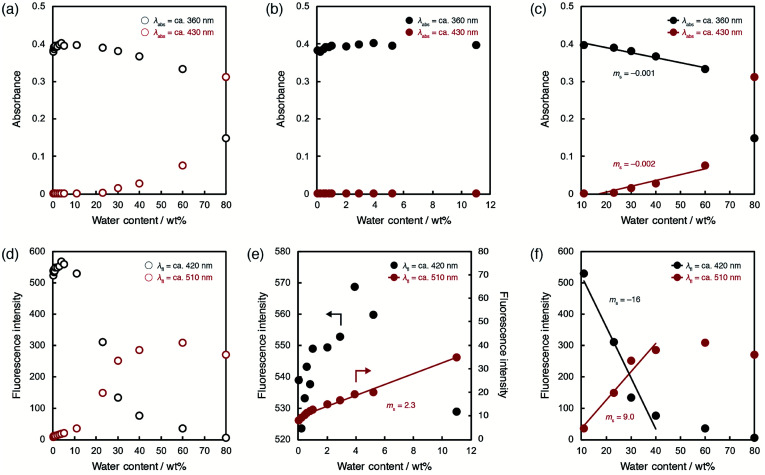
Peak intensities of photoabsorption bands at around 360 and 430 nm in acetonitrile solutions of ET-1 with (a) 0.0265–80 wt%, (b) 0.0265–11 wt%, and (c) 11–80 wt% of water. Peak intensities of fluorescence bands at around 420 and 510 nm (*λ*^ex^ = 302 nm) in acetonitrile solutions of ET-1 with (d) 0.0265–80 wt%, (e) 0.0265–11 wt%, and (f) 11–80 wt% of water.

**Fig. 6:**
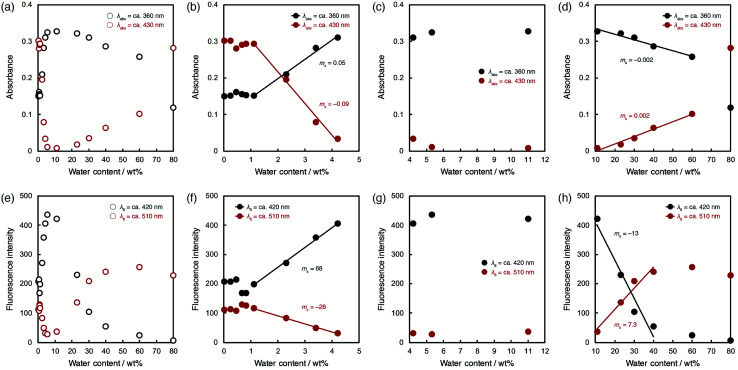
Peak intensities of photoabsorption bands at around 360 and 430 nm in acetonitrile solutions of ET-1-BF_3_ with (a) 0.0201–80 wt%, (b) 0.0201–4.2 wt%, (c) 4.2–11 wt%, and (d) 11–80 wt% of water. Peak intensities of fluorescence bands at around 420 and 510 nm (*λ*^ex^ = 302 nm) in acetonitrile solutions of ET-1-BF_3_ with (e) 0.0201–80 wt%, (f) 0.0201–4.2 wt%, (g) 4.2–11 wt%, and (h) 11–80 wt% of water.

To evaluate the optical sensing ability in detail, the peak intensities of photoabsorption and fluorescence bands were plotted against the water content in acetonitrile solutions of ET-1 (Fig. 5) and ET-1-BF_3_ (Fig. 6). In the case of ET-1, although the maximum intensities of the photoabsorption bands at around 360 (*A*_360_) and 430 (*A*_430_) nm were almost unchanged upon increase in water content below 11 wt% (Fig. 5b), *A*_360_ decreased and *A*_430_ increased linearly as a function of water content in the range of 11–60 wt% (Fig. 5c). The slopes (*m*_s_) in the plots and the correlation coefficient values (*R*^2^) for the calibration curves are −0.001 and 0.97 for *A*_360_ and 0.002 and 0.91 for *A*_430_, respectively. On the other hand, the maximum intensities of the fluorescence bands at around 420 (FL_420_) and 510 (FL_510_) nm increased with increase in the water content even below 11 wt% due to the formation of ET-1-H_2_O (Fig. 5e). The *m*_s_ and *R*^2^ values for FL_510_ are 2.3 and 0.99, respectively, which show good response and linearity. Moreover, the plots of FL_420_ and FL_510_ show significant decrease and increase, respectively, with increasing water content above 11 wt% due to the generation of ET-1-H^+^ (Fig. 5f). The linear relationships were also observed between the fluorescence intensities (FL_420_ and FL_510_) and water content in the 11–40 wt% range. The *m*_s_ and *R*^2^ values are −16 and 0.95 for FL_420_ and 9.0 and 0.95 for FL_510_, respectively. These results, *i.e.*, the linear relationships observed over the wide range of water content, suggest that the precise determination of water in organic solvent is possible over a wide range by using a small amount of ET-1 as an optical sensor.

ET-1-BF_3_ showed distinctly different behavior from ET-1 below 11 wt% of water content (Fig. 6). The photoabsorption and fluorescence intensities drastically changed with increasing water content, although thresholds were observed at around 1 wt% (Fig. 6b and f). The slow release of BF_3_ and incomplete conversion within the reaction time (1 day) would cause the appearance of the thresholds. Indeed, the thresholds disappeared in the plots obtained using the solutions stored in the dark for 2 days (Fig. S1, ESI†). In the water content range of 1.1–4.2 wt%, *A*_360_ increased but *A*_430_ decreased in a linear fashion with increase of water content (Fig. 6b). The *m*_s_ and *R*^2^ values are 0.05 and 0.99 for *A*_360_ and −0.09 and 0.99 for *A*_430_, respectively. Similar contrasting behavior were observed between the plots of FL_420_ and FL_510_ in the low content range with linear relationships (Fig. 6f). The large *m*_s_ values (68 for FL_420_ and −28 for FL_510_) and the *R*^2^ values close to 1 (0.99 for FL_420_ and FL_510_) indicate a superior performance (sensitivity and accuracy) of ET-1-BF_3_ as a water sensor, although the presence of thresholds precludes estimation of the detection limits. In the 4.2–11 wt% range, only slight changes were observed in the photoabsorption and fluorescent intensities (Fig. 6c and g). However, in the higher water content range of 11–60 wt%, *A*_360_ and *A*_430_ distinctly changed (*m*_s_ = −0.002 and 0.002, respectively) with linear relationships (*R*^2^ = 0.96 and 0.98, respectively) due to the formation of ET-1-H^+^ (Fig. 6d), as is the case for ET-1 (Fig. 5c). Moreover, FL_420_ decreased and FL_510_ increased linearly as a function of the water content in the 11–40 wt% range (*m*_s_ = −0.002 and 0.002 and *R*^2^ = 0.95 and 0.96, respectively) (Fig. 6h). These values show good agreement with those observed in the ET-1 solutions (Fig. 5f). Therefore, the complexation of ET-1 with BF_3_ could significantly tune and improve the sensitivity and response, particularly in the low water content range.

**Fig. 7:**
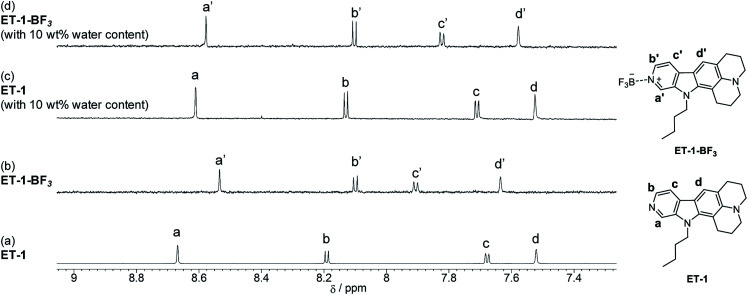
^1^H NMR spectra of (a) ET-1 and (b) ET-1-BF_3_ in CD_3_CN and (c) ET-1 and (d) ET-1-BF_3_ in CD_3_CN containing 10 wt% of D_2_O (*c* = 1.0 × 10^−4^ M).

**Fig. 8:**
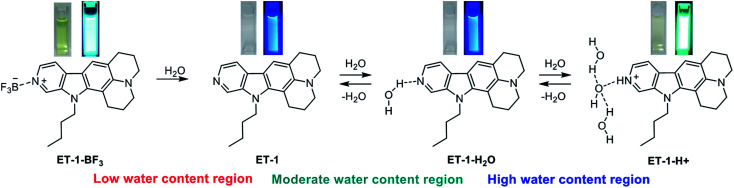
Proposed mechanisms for the detection of water in organic solvent by the colorimetric and fluorescent sensor ET-1-BF_3_. Photographs are color (left) and fluorescence (right) images of the acetonitrile solutions with various amounts of water.

To elucidate the mechanisms for the detection of water, ^1^H NMR spectra of ET-1 and ET-1-BF_3_ in CD_3_CN with and without 10 wt% of deuterated water were obtained (Fig. 7). The complexation with BF_3_ significantly shifted the peaks of the aromatic protons, H_a_ and H_b_, in ET-1 to the higher magnetic field and those of the aromatic H_c_ and H_d_ to the lower field, while the aliphatic protons were affected little (Fig. 7a and b).^59^ The addition of 10 wt% of D_2_O to the ET-1 solution, which generates ET-1-D_2_O as discussed above, caused large upfield peak shifts of H_a_ and H_b_ close to the pyridinic nitrogen atom, a slight downfield shift of H_c_, and no shift of H_d_ distant from the nitrogen atom (Fig. 7c). These changes are similar but lesser extents to the BF_3_ complexation. In the ET-1-BF_3_ solution with 10 wt% of D_2_O, the H_d_ peak shifted to the position close to those observed in the ET-1 solutions with and without 10 wt% of D_2_O (Fig. 7a and c), indicating the release of some BF_3_ (Fig. 7d). The appearance of the H_a_, H_b_, and H_c_ peaks between those of the ET-1 solution containing 10 wt% of D_2_O and the ET-1-BF_3_ solution without D_2_O also supports the partial release. Therefore, the ET-1-BF_3_ solution containing 10 wt% of D_2_O can be considered to be a mixture of ET-1-BF_3_, ET-1, and ET-1-D_2_O. Based on the results obtained in the photoabsorption, fluorescence, and ^1^H NMR measurements and previous studies,^41,42,50–59^ we proposed plausible mechanisms for the detection of water in organic solvent using ET-1-BF_3_ in Fig. 8. In the region of low water content, a number of complex release BF_3_ below *ca.* 10 wt%,^41,42^ and the resultant ET-1 starts forming the hydrogen-bonded complex (ET-1-H_2_O) from *ca.* 1–2 wt%.^50–58^ The ET-1-H_2_O forms over a wide range of water content. In the region of high water content above *ca.* 10 wt%, the hydrogen-bonded PTC (ET-1-H^+^) gradually generates.^50–58^ Previously, a photochromic dye sensor with intramolecular hydrogen bonding that can detect hydrogen bonding characters of media has been developed.^31^ET-1 detects water based on hydrogen bonding with water molecules similarly to the photochromic sensor. However, differently from the sensor, ET-1 stepwisely forms two complexes hydrogen-bonded with one water molecule or several water molecules, as the precursor 9-MP does.^50–58^ Indeed, the responses of ET-1-BF_3_ to water were demonstrated visually as shown in Fig. 8. The yellow color of the ET-1-BF_3_ solution faded away with increasing water content due to the release of BF_3_ and subsequent formation of ET-1-H_2_O, followed by the restoration of the yellow color as a result of the ET-1-H^+^ formation. The fluorescence color also changed in the order of light blue, blue, and green, together with the color changes.

## Conclusions

We have developed ICT-type colorimetric and fluorescence sensors, ET-1 and ET-1-BF_3_, for detection and determination of water over a wide range of the concentration in organic solvent. The sensors can response to water differently depending on the content. In the range of low water content (below *ca.* 10 wt%), ET-1-BF_3_ releases BF_3_ to generate ET-1, and ET-1 forms the hydrogen-bonded complex with one water molecule (ET-1-H_2_O). In higher water contents (above *ca.* 10 wt%), the hydrogen-bonded PTC (ET-1-H^+^) gradually generates. Compared with the previous ICT-type sensors with β-carboline structure (9-MP and 9-MP-BF_3_), the sensitivity and spectral response to water are significantly improved over a wide concentration range by the fusion with the electron-donating juloidine and complexation with BF_3_. The juloidine conjugation enhances the basicity of the pyridinic nitrogen atom and the ICT characteristic. The BF_3_ complexation contributes to the improvements particularly in the low water contents. This work shows a useful approach to tuning and improvement of optical sensors for sensitive detection and precise determination of water over various concentrations.

## Conflicts of interest

There are no conflicts to declare.

## Supplementary Material

RA-009-C9RA07136A-s001
