# Supplementary material for: Development of an intramolecular charge transfer-type colorimetric and fluorescence sensor for water by fusion with a juloidine structure and complexation with boron trifluoride
Source: RSC Adv. 2019 Oct 3;9(54):31466–73. doi: 10.1039/c9ra07136a (PMC9072393; doi:10.1039/c9ra07136a)
Supplement: RA-009-C9RA07136A-s001 [file RA-009-C9RA07136A-s001.pdf]

Electronic Supplementary Material (ESI) for RSC Advances.
This journal is © The Royal Society of Chemistry 2019

Supplementary Information

Development of an intramolecular charge transfer-type colorimetric and fluorescence sensor for water by fusion with juloidine structure and complexation with boron trifluoride

Keiichi Imato, Toshiaki Enoki and Yousuke Ooyama*

Department of Applied Chemistry, Graduate School of Engineering, Hiroshima University, Higashi-Hiroshima, 739-8527, Japan.

Fax: +81 824 24 5494; Tel: +81 824 24 7689; E-mail:yooyama@hiroshima-u.ac.jp

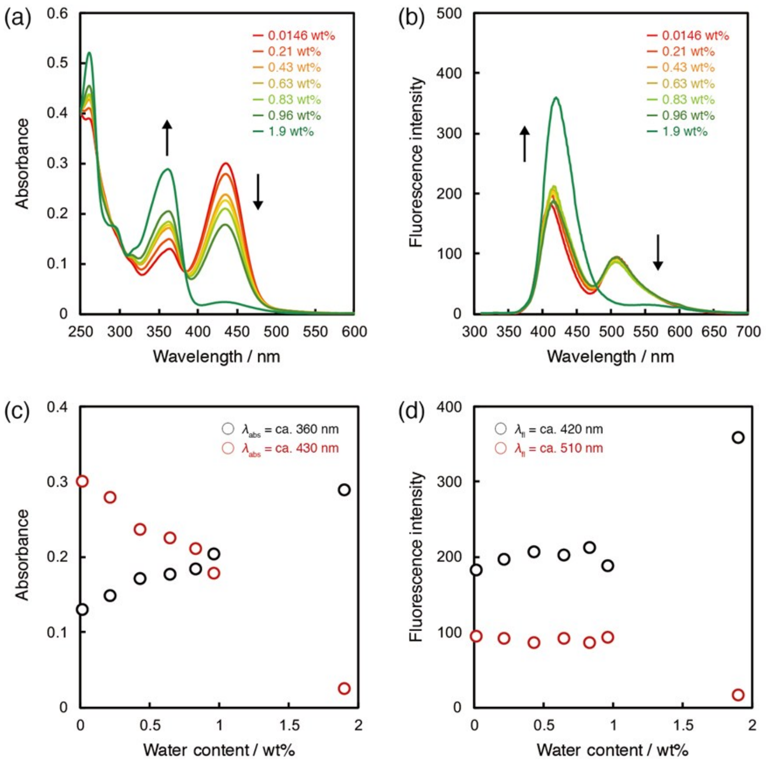

Fig. S1 (a) Photoabsorption and (b) fluorescence spectra of **ET-1-BF$_3$** (c = 2.0 × 10^{-5} M) in acetonitrile containing 0.0146–1.9 wt% of water. Prior to the measurements, the solutions were stored in the dark for 2 days after addition of water. Peak intensities of (c) absorption bands at around 360 and 430 nm and (d) fluorescence bands at around 420 and 510 nm (λ^{ex} = 302 nm) in acetonitrile solutions of **ET-1-BF$_3$** with 0.0146–1.9 wt% of water.

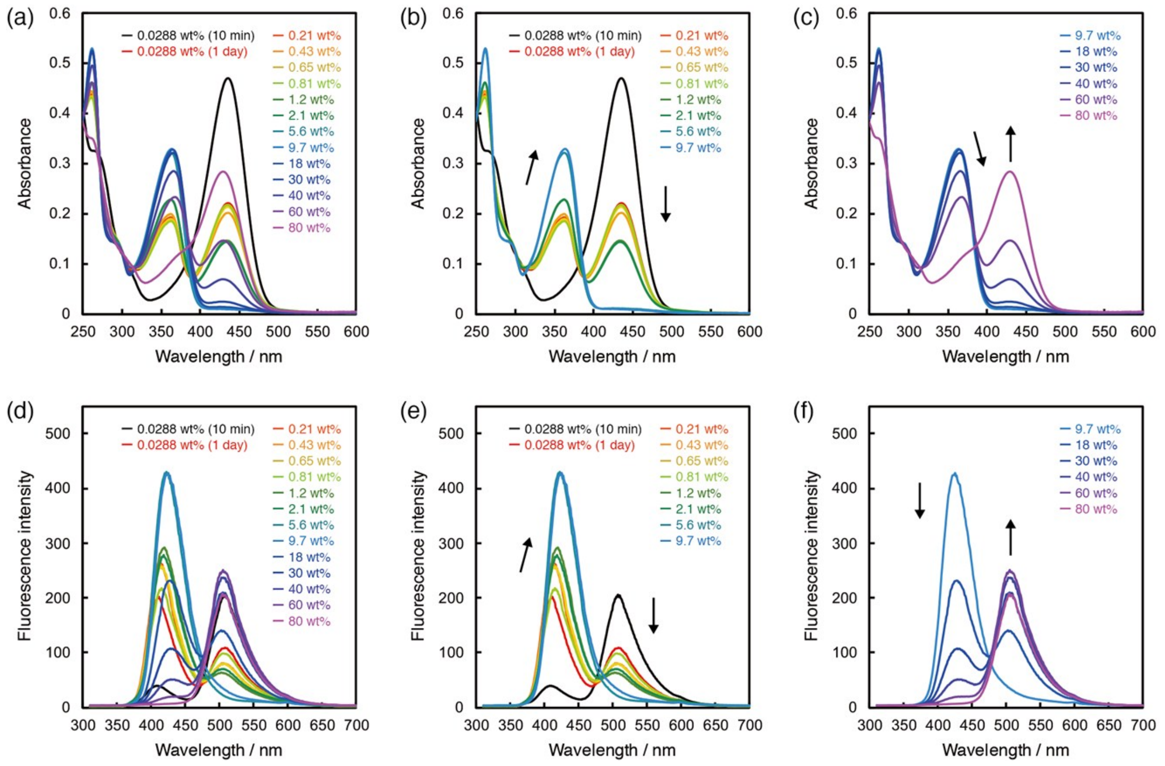

Fig. S2 Results of another solution for demonstration of reproducibility. (a) Photoabsorption spectra of **ET-1-BF**$_3$ (c = 2.0 × 10^{-5} M) in acetonitrile containing (a) 0.0288–80 wt%, (b) 0.0288–9.7 wt%, and (c) 9.7–80 wt% of water. Fluorescence spectra of **ET-1-BF**$_3$ (c = 2.0 × 10^{-5} M, λ^{ex} = 302 nm) in acetonitrile containing (d) 0.0288–80 wt%, (e) 0.0288–9.7 wt%, and (f) 9.7–80 wt% of water.

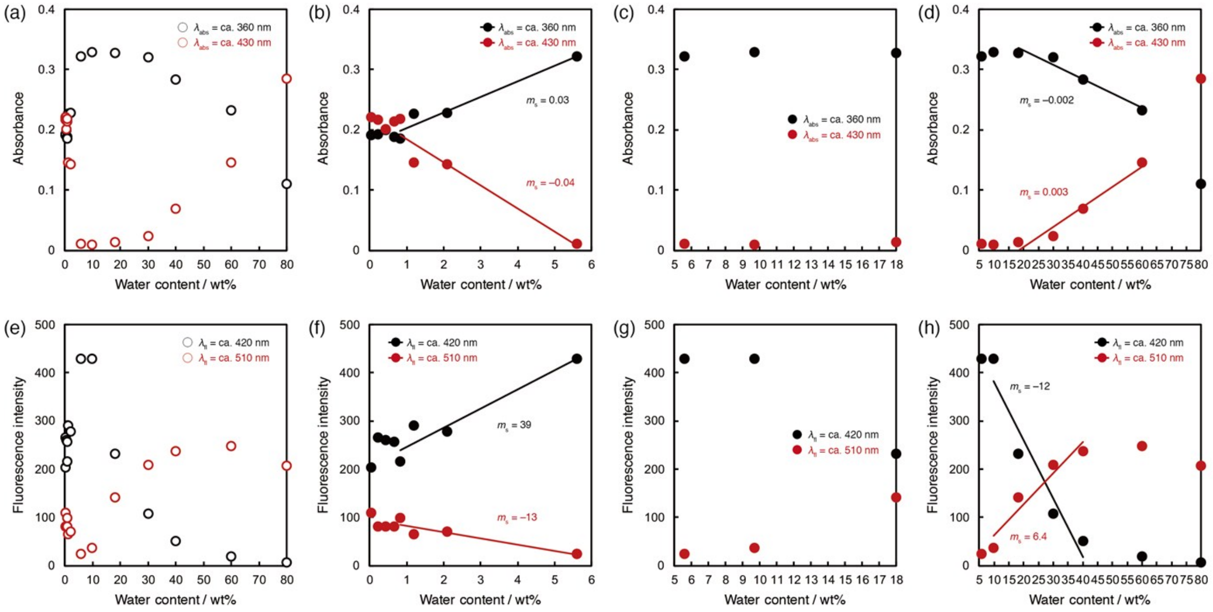

Fig. S3 Results of another solution for demonstration of reproducibility. Peak intensities of photoabsorption bands at around 360 and 430 nm in acetonitrile solutions of **ET-1-BF$_3$** with (a) 0.0288–80 wt%, (b) 0.0288–5.6 wt%, (c) 5.6–18 wt%, and (d) 5.6–80 wt% of water. Peak intensities of fluorescence bands at around 420 and 510 nm (λ^{ex} = 302 nm) in acetonitrile solutions of **ET-1-BF$_3$** with (e) 0.0288–80 wt%, (f) 0.0288–5.6 wt%, (g) 5.6–18 wt%, and (h) 5.6–80 wt% of water.

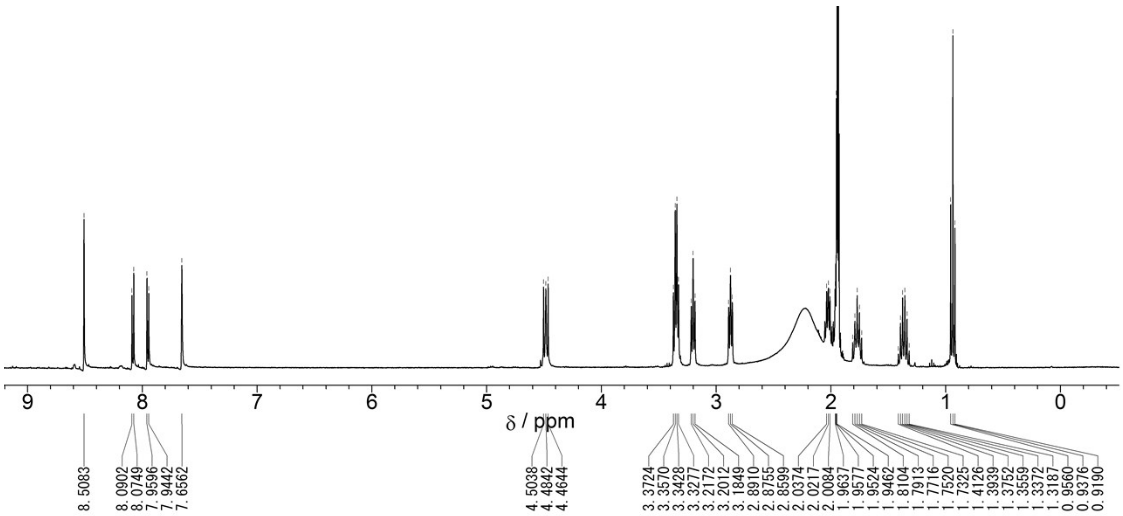

Fig. S4 ^{1}H NMR spectrum of **ET-1-BF$_3$** (CD_3CN).

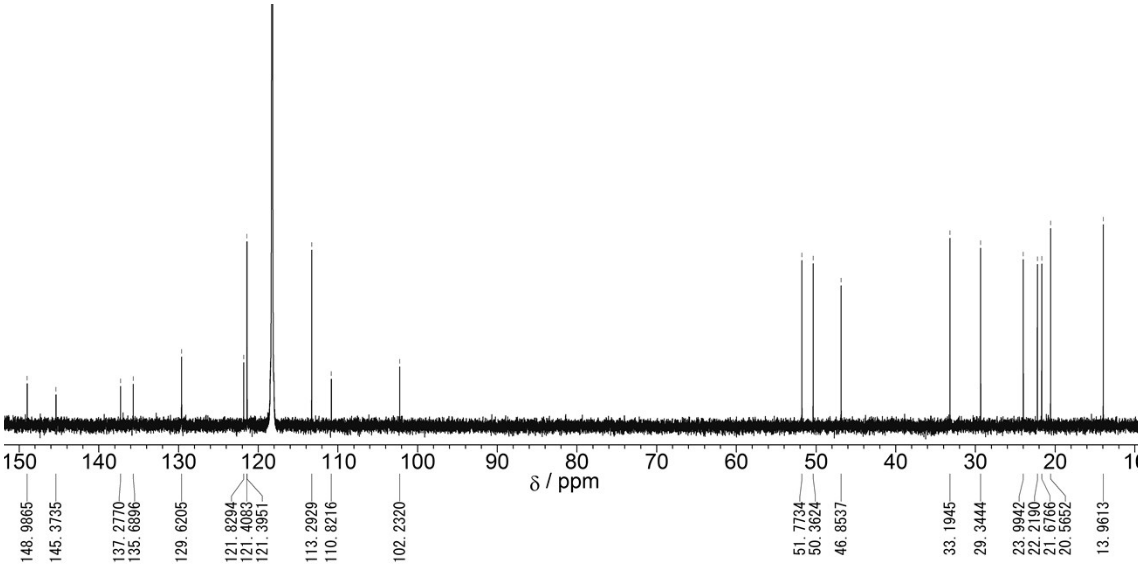

Fig. S5 ^{13}C NMR spectrum of **ET-1-BF$_3$** (CD_3CN).

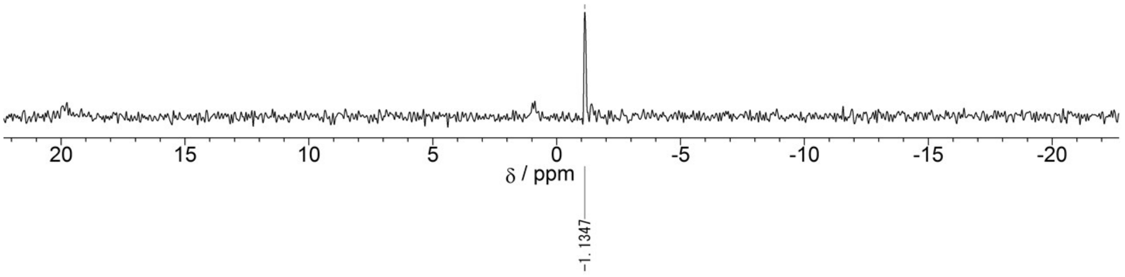

Fig. S6 ^{11}B NMR spectrum of **ET-1-BF$_3$** (CD_3CN).
